# Annotations

**Published:** 1909-05-15

**Authors:** 


### ANNOTATIONS.

### Tubercle Bacilli in the Blood.

Less than three months ago a paper appeared in one of the most influential and widely circulated trans-Atlantic medical journals, whose author claimed to be able to demonstrate tubercle bacilli in films of blood taken from patients suffering from tuberculosis. This bacteriæmia he believed to occur not only in generalised miliary tuberculosis, but in all cases of localised active disease. Upon the face of it such a discovery has bearings whose importance can scarcely be overestimated—a sure and certain method of diagnosis so simple, so painless, so devoid of possible ill-effects would revolutionise existing methods completely. Nor is there anything essentially incredible about the possibility. Tubercle bacilli must obviously sometimes be circulating in the blood, even during health; so that no hypothetical reason exists why they should not constantly do so when an active manufactory exists somewhere in the body. The point that requires elucidation, however, is whether tubercle bacilli can actually always be found in the blood-stream; whether as a fact their detection is always possible in a tuberculous patient. Upon this very definite question it would seem, according to yet another American journal of the highest repute, that the original investigator who committed himself to a positive answer is wrong. At two large New York hospitals special investigations have been made, the result of which is absolutely to negative that contention; and some very sweeping criticisms are passed upon the unscientific way in which the original experiments were conducted. There can be no valid reason why similar control investigations should not be made in this country; and such independent research would have the added value of being quite free from any possible imputations of professional jealousy, as well as tending to decide finally a point of the most far-reaching importance.

### Chlorodyne and the Poison Schedule.

Notwithstanding the care with which the Poisons and Pharmacy Bill was discussed before it became law, it seems that there are still possibilities for persons to obtain poisonous quantities of certain drugs without infringing the Act. This has been brought out by a recent inquest, held by the City Coroner, Dr. F. J. Waldo, concerning the death of Emily Landsell, aged 43, the wife of a publican. It is true that the jury returned a verdict of "Accidental Death," but it seems equally true that death was caused by chlorodyne poisoning. According to the evidence, Mrs. Landsell was found dead in bed, and it was stated that she was in the habit of taking chlorodyne in order to induce sleep. One doctor said that death was due to heart failure, accelerated by alcoholism; but the police divisional surgeon attributed death to chlorodyne poisoning. Owing to this difference of medical opinion, expert toxicological evidence was sought from Dr. Womack, who pronounced for morphine poisoning, probably due to the woman having taken about half an ounce of chlorodyne, which would contain, it seems, about one grain of morphine. It would appear that chemists have received notice that they are not obliged to request purchasers of chlorodyne to sign the poisons book, as that medicine does not come within Part I. of the Poisons Schedule. Seeing, therefore, that chlorodyne may cause death, it is a matter of some importance for medical men to know how it escapes the Act of 1908, especially as it appears that chlorodyne may sometimes contain another poison besides morphine—namely hydrocyanic or prussic acid, though in some similar preparations this is replaced by tincture of capsicum. Under the new Act 0.1 per cent. of prussic acid or more brings preparations containing it under Part I. of the Scedule. The morphine in a mixture must reach 1 per cent. for that mixture to come under Part I. of the Schedule. The chlorodyne in question in the case of Emily Landsell contained, we are told, only 1 grain of morphine in 4 drachms, and thus, though fatal in its effects, it entirely escaped the Act.

### Art and Morals.

In a letter recently contributed to a lay contemporary a lady complained of the "great injustice" which was contemplated by those who were attempting to raise thousands of pounds to buy the Duke of Norfolk's Holbein, while St. Bartholomew's Hospital is unable to pay its way. "Surely," she says, "the Government would do more good to help obtain medical and surgical attendance for the poor than buy a picture which, at the very best, would only give enjoyment to a limited number." We confess ourselves in general agreement with the correspondent, in the limited sense that the proposed contribution of the State seems totally disproportionate in amount to the degree of satisfaction, or moral improvement, which is likely to accrue to the public from the expenditure. It is a *bourgeois* admission, and we lay ourselves open to the scorn of æsthetes; yet many will be bound to agree that there is a deal of clap-trap talked about the elevating influence of old masters: clap-trap we say, because the mere technical beauties of first-rate paintings are unappreciable by an infinite majority, and no amount of "high-falutin'" will alter the fact. It is not as though the picture in question speaks any lesson of patriotism, raises any historic memories of which we are proud, or inspires any exaltation of morals. It is a portrait of a woman, executed by a foreigner long dead, and of no conceivable advantage to the nation except as a museum specimen of mastery in technical art. Naturally, from a sentimental point of view, it is gratifying to this country to possess a fine museum of such things, but one feels less prepared for a sacrifice with such an object in view than one would be were the object a real "elevation" of the people such as we hear so much about. Touching the alternative suggested by the writer of the letter, we prefer to keep an open mind. It may be that State support of hospitals will be needed in the future, but it is not yet so needed owing to the amazing charity of our public, and we do not doubt that St. Bartholomew's Hospital will weather its temporary embarrassments without it.

